# Loratadine versus levocetirizine in chronic idiopathic urticaria: A comparative study of efficacy and safety

**DOI:** 10.4103/0253-7613.62399

**Published:** 2010-02

**Authors:** P. Anuradha, Rituparna Maiti, J. Jyothirmai, Omer Mujeebuddin, M. Anuradha

**Affiliations:** Department of Pharmacology, Prathima Institute of Medical Sciences, Nagunur Road, Karimnagar, Andhra Pradesh, India; 1 Department of Dermatology, Prathima Institute of Medical Sciences, Nagunur Road, Karimnagar, Andhra Pradesh, India

**Keywords:** Absolute eosinophil count, chronic idiopathic urticaria, levocetirizine, loratadine, total symptom score

## Abstract

**Background:**

Treatment of chronic idiopathic urticaria (CIU) is challenging because of its unpredictable course and negative influence on the quality of life. New treatments are being developed, but antihistaminics remain the cornerstone of the therapeutic approach. Newer generation antihistaminics such as loratadine and levocetirizine have already proved to be safe and efficacious for CIU.

**Objective:**

To choose the better drug between loratadine and levocetirizine for CIU, by comparing their efficacy and safety.

**Methods:**

A randomized, open, outdoor-based clinical study was conducted on 60 patients of CIU, to compare the two drugs. After initial clinical assessment and baseline investigations, loratadine was prescribed to 30 patients and levocetirizine to another 30 patients for four weeks. At follow-up, the patients were re-evaluated and then compared using different statistical tools.

**Result:**

The comparative study showed that the changes in differential eosinophil count (*P* = 0.006) and absolute eosinophil count (*P* = 0.003) in the levocetirizine group was statistically significant. The results of the Total Symptom Score showed better symptomatic improvement of CIU with levocetirizine as compared to loratadine. The overall incidence of adverse drug reactions was also found to be less in the levocetirizine group.

**Conclusion:**

An analysis of the results of all the parameters of safety and efficacy proves the superiority of levocetirizine over loratadine for CIU.

## Introduction

Urticaria is a transient vascular reaction pattern characterized by circumscribed, edematous, itchy lesions, usually lasting for a few hours to one or two days. In particular, chronic urticaria is characterized by the occurrence of wheals daily or almost daily for a period of at least six weeks, with an estimated lifetime prevalence of 0.5% across all populations studied.[1,2] Despite an exhaustive and expensive diagnostic approach, searches for the etiology of chronic urticaria are mostly frustrating, because in most cases the causative agent is unknown. In these cases a diagnosis of chronic idiopathic urticaria (CIU) is made.[3]

CIU is a distressing condition that severely affects the patients' quality of life and performance. Effective treatment is thus required in all cases, where avoidance of eliciting factors is not feasible. General practitioners and specialists have to deal with the challenge of treating the symptoms and ensuring a decent quality of life for their patients. In the last few decades an increasing understanding of the pathomechanisms involved in urticaria has highlighted the heterogeneity of different subtypes. Clarity of nomenclature is required not only to choose the correct measures in diagnosis and management, but also to compare data from different studies.[4]

New treatments are being developed, but antihistaminic drugs remain the cornerstone of the therapeutic approach. During recent years, new antihistaminics such as loratadine and levocetirizine have been marketed and indicated for the treatment of urticaria, with the benefit of a better safety profile.[2,3,5–11]

Although individually loratadine and levocetirizine are efficacious for CIU, unique findings in some studies indicate a likelihood of differences between these two drugs.[9,12–16] Therefore, this study has been conducted to compare the therapeutic efficacy and tolerability of levocetirizine and loratadine in patients suffering from CIU. The relative absence of data in this domain is an incentive to further explore this aspect of the disease.

## Materials and Methods

The present study is a randomized, open, comparative clinical study between levocetirizine and loratadine for CIU. Procedures followed in this study are in accordance with the ethical standards laid down by ICMR's Ethical Guidelines for Biomedical Research on Human Subjects (2006), with prior permission from the Institutional Ethics Committee.

### Subjects

Patients (n = 60) between the ages of 12 and 60 years, suffering from CIU, were recruited from the Dermatology Outpatient Department of Prathima Institute of Medical Sciences, Karimnagar, Andhra Pradesh, India. Patients suffering from other forms of urticaria, with significant concomitant illness (e.g., malignancies or hepatic, psychiatric, endocrine or other major systemic diseases), pregnant women, lactating mothers, females on oral contraceptive pills, patients on antihistaminic therapy for 72 hours, or steroids for one month, were excluded from the study. Certain special tests, such as, test for dermographism, ice-cube test, and exercise test were carried out in selected patients, as suggested by the history of their illness, to rule out other forms of chronic urticaria. A written informed consent was taken from all the patients included in the trial after explaining the patient's diagnosis, the nature and purpose of the proposed treatment, the risks and benefits of the proposed treatment (loratadine / levocetirizine), alternative treatment (corticosteroids), and the risks and benefits of the alternative treatment. After systematic randomization, 60 patients who participated in the study were divided into two groups; 30 patients were assigned to receive loratadine, 10 mg daily, and 30 patients received levocetirizine 5 mg daily, for a period of four weeks. The patients received the drugs free of cost from our institute pharmacy. At the first visit, selected cases of CIU were thoroughly interviewed, individually, to record the circumstances that precipitated the attacks and a detailed history was taken on baseline symptomatology. The vital signs were measured as, routine clinical check up. Physical examinations, especially dermatological tests, the size of the wheals were measured, and baseline clinical investigations were carried out. At the four-week follow-up, a physical examination and baseline investigations were repeated and all post drug symptoms were recorded.

### Laboratory measurements

Routine analysis of blood [Total Leucocyte Count (TLC), Differential Count (DC), and Absolute Eosinophil Count (AEC)] was estimated in each patient at baseline (First Visit) and at follow-up (Second Visit).

### Efficacy measures

All patients were evaluated for the degree of pruritus, size of wheals, number of wheals, and number of separate urticarial episodes. At both visits, evaluations were made for each patient by the same investigator.

Efficacy measures were scored according to the following scales: Pruritus: 0 (none), 1 (mild), 2 (moderate), and 3 (severe); Number of wheals: 0 (none), 1 (1 – 10 wheals), 2 (11 – 20 wheals), 3 (> 20 wheals); Size of wheals (mean diameter): 0 (no lesion), 1 (< 1.27 cm), 2 (1.27 – 2.54 cm), 3 (> 2.54 cm); Number of separate urticarial episodes: 0 (no episodes), 1 (1 episode), 2 (2 – 3 episodes), 3 (> 3 episodes). The maximum value of the total symptoms score (TSS) was 12.[3]

### Safety measures

Safety and tolerability were assessed on the basis of the adverse events reported, or by comparing the baseline symptoms with post-drug symptoms, or changes in vital signs and physical examination findings recorded before and at the end of treatment.

### Statistical Analysis

Statistical analysis was carried out by Paired t-test / Wilcoxon Signed Rank test, t-test / Mann Whitney Rank Sum test, and Fisher's Exact test, using statistical software Jandel SigmaStat version 2. Interval data have been expressed as Mean ± SD and categorical data in percentage. A *P* value of <0.05 was considered as statistically significant.

## Results

The two groups were homogenous with respect to baseline demographic data, including patients' age and sex, duration of disease, and severity [Table 1]. At follow up, nine were lost and a total of 51 patients (26 in the loratadine group and 25 in the levocetirizine group) completed the trial. Among nine patients, six patients did not report for follow-up and three patients were non-compliant with the treatment. The percentages of the female patients were 60 and 56.7 in the loratadine and levocetirizine groups, respectively. The mean age of the patients was 33.4 and 34.8 years and the patients were symptomatic for a mean duration of 10.7 and 9.6 weeks in the loratadine and levocetirizine groups, respectively.

**Table 1:** Baseline demographic data and clinical characteristics of the patients of chronic idiopathic urticaria

*Characteristics*	*Loratadine group*	*Levocetirizine group*	*P value*@
Number of patients recruited	30	30	
Number of patients at follow-up	26	25	
Female sex (%)	60	56.7	
Age (years)	33.43 ± 12.23	34.80 ± 12.16	0.67
Duration of CIU (weeks)	10.7 ± 2.9	9.6 ± 2.1	0.13
Total leucocyte count	8420 ± 1095	8678 ± 1024	0.34
DC neutrophil (%)	63.1 ± 4.7	63.7 ± 4.3	0.65
DC lymphocyte (%)	32.6 ± 4.1	34.8 ± 4.9	0.07
DC eosinophil (%)	4.1 ± 1.5	4.2 ± 1.3	0.57
Absolute eosinophil Count	350.5 ± 164.7	377.7±127.7	0.19
Total Symptom Score	7.8 ± 1.9	7.3 ± 2.2	0.23

Data are in Mean ± SD

@ Analysis: by Student's ‘t’-test

### Change in Total Leucocyte Count

There was a 2.1% decrease in TLC in the loratadine group in comparison to 2.9% in the levocetirizine group. The change in the loratadine group was not statistically significant (*P* = 0.07), but there was a significant decrease (*P* = 0.004) in the levocetirizine group. When the changes in the two groups were compared using the Mann Whitney Rank Sum test, the change in the levocetirizine group was found to be statistically non-significant (*P* = 0.41) [Table 2].

**Table 2:** Comparison of different variables studied before and after treatment in both groups, among follow-up cases, in the comparative study between loratadine and levocetirizine

*Variable*	*Loratadine group*				*Levocetirizine group*				*Difference between the groups [Δ% Loratadine group vs. Δ% Levocetirizine group] Ψ*
			
	*1^st^ Visit*	*2^nd^ Visit*	*% change*	*P value Ω*	*1^st^ Visit*	*2^nd^ Visit*	*% change*	*P value Ω*		
Total leucocyte count	8485 ± 1108	8277 ± 935	2.1	0.070	8690 ± 1067	8408 ± 838	2.9	0.004*	0.41
DC neutrophil (%)	63.5 ± .8	62.5 ± 3.9	1.0	0.100	64.2 ± 4.5	61.8 ± 3.1	2.4	0.003*	0.06
DC lymphocyte (%)	32.4 ± 4.3	34.3 ± 3.4	1.9	0.003*	34.7 ± 5.3	37.2 ± 3.6	2.5	< 0.001*	0.96
DC eosinophil (%)	4.12 ± 1.63	3.69 ± 1.38	0.43	0.021*	4.24 ± 1.39	3.12 ± 1.33	1.12	< 0.001*	0.006*
Absolute eosinophil count	357 ± 176	310 ± 134	7.74	0.004*	368 ± 131	262 ± 116	27.89	< 0.001*	0.003*
Total symptom score	7.85 ± 1.99	7.46 ± 1.92	4.85	0.002*	7.32 ± 2.29	6.24 ± 1.74	13.32	< 0.001*	< 0.001*

Data are in mean ± SD, Ω Paired t-test / wilcoxon signed rank test, Ψ t-test / mann whitney rank sum test

* Statistically significant

### Change in differential count of neutrophil and lymphocyte

There was a 1.0% decrease in neutrophils and 1.9% increase in lymphocytes in the loratadine group and a 2.4% decrease in neutrophils and 2.5% increase in lymphocytes in the levocetirizine group. The changes in the levocetirizine group were statistically significant, but when the changes in the two groups were compared by the Mann Whitney Rank Sum test, the change in the levocetirizine group was found to be statistically non-significant (*P* = 0.06 for DC neutrophil and *P* = 0.96 for DC lymphocyte) [Table 2].

### Change in differential count of eosinophil

There was a 0.43% decrease in eosinophils in the loratadine group in comparison to 1.12% in the levocetirizine group. The changes in both the loratadine and levocetirizine groups were statistically significant and when the changes in the two groups were compared by the Mann Whitney Rank Sum test, the change in the levocetirizine group was found to be statistically significant as compared to the loratadine group (*P* = 0.006) [Table 2].

### Change in absolute eosinophil count (AEC)

There was a 7.74% decrease in the Absolute Eosinophil Count (AEC) in the loratadine group as compared to a 27.89% decrease in the levocetirizine group. The changes in both the loratadine and levocetirizine groups were statistically significant and when the changes in the two groups were compared by the Student's t-test, the change in the levocetirizine group was found to be statistically significant (*P* = 0.003) [Table 2].

### Efficacy analysis: Change in total symptom score (TSS)

Total Symptom Score (TSS) was calculated for the patients of both groups on their first and second visits. There was a 4.85% decrease in TSS in the loratadine group as compared to 13.32% in the levocetirizine group. The changes in both the loratadine and levocetirizine groups were statistically significant and when the changes in the two groups were compared by the Mann Whitney Rank Sum test, the change in the levocetirizine group was found to be statistically significant (*P* < 0.001) [Table 2].

It was found that in the loratadine group, among 26 follow-up cases, TSS improved in 10 patients but in the levocetirizine group, among 25 patients, TSS improved in 18 patients. This finding has been represented in a 2 × 2 contingency table and the statistical significance was tested by the Fisher's Exact test (Fisher's Exact test: *P* = 0.025). *P* < 0.05 indicates that the change in the levocetirizine group was statistically significant and it was not by random occurrence.

**Figure 1:**
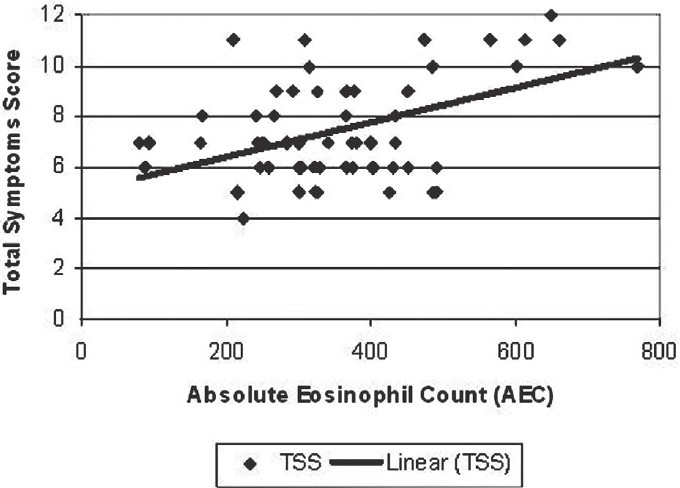
Correlation between total symptom score (TSS) and absolute eosinophil count (AEC) at first visit of the patients of CIU, who participated in the comparative study between loratadine and levocetirizine. [Correlation coefficient = 0.48, *P* < 0.001]

Absolute Eosinophil Count bears a significant positive correlation (Coefficient of Correlation = 0.48, *P* < 0.001) with TSS. The trendline in the scatter diagram [Figure 1] shows an increase in AEC with an increase in TSS.

### Safety analysis

No clinically significant changes in vital signs, laboratory parameters or physical examination were observed during the study in any group. In the loratadine group, out of five patients who experienced adverse effects, two complained of drowsiness, one had headache, one had gastric irritation, and one patient had dryness of mouth. In the levocetirizine group three patients complained of drowsiness. The overall incidence of adverse effects was 19.2 and 12% in the loratadine and levocetirizine groups, respectively. To compare the incidence of adverse effects of the two groups, the Fischer's Exact test was performed and it was found to be statistically non-significant (*P* = 0.70).

## Discussion

Although symptomatic treatment of CIU and ensuring a good quality of life for the patients is challenging to the physicians, an increasing understanding of the pathophysiological mechanisms in the last few decades has revealed the potential of a new generation of antihistaminics for the treatment of this condition. Loratadine and levocetirizine have already proved their benefit individually, with a better safety profile, in several clinical trials, but this is the first study to compare their efficacy and safety and to thereby choose the better agent of the two.

The baseline data shows that there is statistically no significant difference between the study groups with respect to the demographic and clinical parameters. This proves the homogeneity of our study subjects in the two groups. The female predominance found in this study supports the previous studies where it has been found that CIU is more common in females.[2,3]

In the present study total leucocyte count, differential count of neutrophils, lymphocytes, eosinophils, and absolute eosinophil count were carried out at both visits and the results were compared between the groups. From the results of Total Leucocyte Count, and differential count of neutrophils and lymphocytes, no conclusion could be made with respect to the superiority of any drug in the improvement of these hematological parameters. However, the comparative changes in the differential count of eosinophils and Absolute Eosinophil Count were found to be significant, and the change in the levocetirizine group was found to be statistically significant as compared to the loratadine group. From these findings it can be concluded that there was a better control of these two routine investigational markers of CIU with levocetirizine as compared to loratadine.

All patients in this study were evaluated for the degree of pruritus, size of wheals, number of wheals, and number of separate urticarial episodes on both the visits. Efficacy measures were scored following a scale where maximum values of the total symptoms score (TSS) was 12. There was a 4.85% decrease in the TSS of the loratadine group, in comparison to 13.32% in the levocetirizine group. The changes in both the loratadine and levocetirizine group were statistically significant and when the changes in the two groups were compared by the Mann Whitney Rank Sum test, the change in the levocetirizine group was found to be statistically significant. The findings in the previous studies on levocetirizine for CIU by Nettis *et al*., Garg *et al*., and Pfaar *et al*., support the findings of this present study.[3,8,11] The Total Symptom Score (TSS) is a widely accepted, standardized, and reliable tool to assess the efficacy of a drug in the treatment of urticaria and a decrease in the scoring suggests that there is an overall clinical improvement in the condition. The comparative changes in TSS in the study groups clearly prove the superiority of levocetirizine over loratadine.

The changes in TSS have been also analyzed by expressing the data in terms of proportions using the Fisher's exact test. The change in the levocetirizine group was statistically significant and it was not by random occurrence. This finding again confirms the better control of the disease with levocetirizine than loratadine.

Although the overall incidence of adverse effects in the levocetirizine group has been found to be lower than in the loratadine group, there was no significant difference between the two groups. The incidence of drowsiness was found to be more in the levocetirizine group, which has been supported by a previous study by Layton *et al*.[12]

The overall superiority of levocetirizine may be attributed to its additional effects such as its ability to decrease serum levels of ELAM-1 (Endothelial Leucocyte Adhesion Molecule-1) and P-selectin or its ability to inhibit resting and GM-CSF (Granulocyte Macrophage-Colony Stimulating Factor) stimulated firm eosinophil adhesion to rhVCAM-1 (recombinant human Vascular Cell Adhesion Molecule-1), under flow conditions.[9,12]

## Conclusion

Analysis of the results of all the parameters of safety and efficacy explores the probable superiority of Levocetirizine over Loratadine for CIU. The findings of this exploratory study can be confirmed by multicentric, randomized, double-blind large population studies.

## Announcements

1. Instructions to authors are available on www.ijp-online.com
  Manuscripts must be submitted online either through www.ijp-online.com (Click “Instructions” and then “Manuscript Submission” and follow the instructions thereafter) or directly through www.journalonweb.com/ijp
2. Subscription details are available from www.ijp-online.com.
3. For non-receipt of journal contact subscriptions@medknow.com or ijp@ijp-online.com
  Please mention your name and affiliation, old/new address and IPS membership number (if available). Institutional subscribers should mention the name and address of the institution and details of the payment made.

**Dr R. K. Dikshit**

Chief Editor

Indian Journal of Pharmacology,

Department of Pharmacology,

B. J. Medical College,

Ahmedabad – 380016.
